# IGFBP3 deposited in the human umbilical cord mesenchymal stem cell‐secreted extracellular matrix promotes bone formation

**DOI:** 10.1002/jcp.26342

**Published:** 2018-03-01

**Authors:** Moyuan Deng, Keyu Luo, Tianyong Hou, Fei Luo, Zhao Xie, Zehua Zhang, Aijun Yang, Bo Yu, Shaoxuan Yi, Jiulin Tan, Shiwu Dong, Jianzhong Xu

**Affiliations:** ^1^ National and Regional United Engineering Lab of Tissue Engineering Department of Orthopaedics Southwest Hospital The Third Military Medical University Chongqing China; ^2^ Department of Biomedical Materials Science College of Biomedical Engineering Third Military Medical University Chongqing China

**Keywords:** bone formation, endogenous MSC, human umbilical cord MSC‐secreted ECM, IGFBP3, recruitment

## Abstract

The extracellular matrix (ECM) contains rich biological cues for cell recruitment, proliferationm, and even differentiation. The osteoinductive potential of scaffolds could be enhanced through human bone marrow mesenchymal stem cell (hBMSC) directly depositing ECM on surface of scaffolds. However, the role and mechanism of human umbilical cord mesenchymal stem cells (hUCMSC)‐secreted ECM in bone formation remain unknown. We tested the osteoinductive properties of a hUCMSC‐secreted ECM construct (*hUCMSC‐ECM*) in a large femur defect of a severe combined immunodeficiency (SCID) mouse model. The *hUCMSC‐ECM* improved the colonization of endogenous MSCs and bone regeneration, similar to the hUCMSC‐seeded scaffold and superior to the scaffold substrate. Besides, the *hUCMSC‐ECM* enhanced the promigratory molecular expressions of the homing cells, including CCR2 and TβRI. Furthermore, the *hUCMSC‐ECM* increased the number of migrated MSCs by nearly 3.3 ± 0.1‐fold, relative to the scaffold substrate. As the most abundant cytokine deposited in the *hUCMSC‐ECM*, insulin‐like growth factor binding protein 3 (IGFBP3) promoted hBMSC migration in the TβRI/II‐ and CCR2‐dependent mechanisms. The *hUCMSC‐ECM* integrating shRNA‐mediated silencing of *Igfbp3* that down‐regulated IGFBP3 expression by approximately 60%, reduced the number of migrated hBMSCs by 47%. In vivo, the *hUCMSC‐ECM* recruited 10‐fold more endogenous MSCs to initiate bone formation compared to the scaffold substrate. The knock‐down of *Igfbp3* in the *hUCMSC‐ECM* inhibited nearly 60% of MSC homing and bone regeneration capacity. This research demonstrates that IGFBP3 is an important MSC homing molecule and the therapeutic potential of *hUCMSC‐ECM* in bone regeneration is enhanced by improving MSC homing in an IGFBP3‐dependent mechanism.

## 1 INTRODUCTION

Large bone defect is a challenge confronting orthopedic surgery. Autologous bone transplantation is the gold standard for bone defect healing, but its availability is limited because of significant comorbidities (Boden, 2000). Bone graft materials with high osteoinductive potentials are the key of large bone defects healing. Tissue engineered technology improves the osteoinductive potential of scaffolds through seeding mesenchymal stem cells (MSCs) on surface of scaffolds (Henkel et al., 2013; Nguyen & Burg, 2015). MSCs secret and deposit substantial quantities of extracellular matrix (ECM) during in vitro culture and ECM modifies the surface of scaffold substrates through decellularizing tissue engineered constructs (Decaris, Mojadedi, Bhat, & Leach, 2012; Harvestine et al., 2016). The MSC‐secreted ECM allows bypassing of living MSC‐induced clinical limits including long‐time storage, economic, and logistic issues (Martin, 2014; Meijer, de Bruijn, Koole, & van Blitterswijk, 2007).

ECM is comprised of rich biological proteins for directing cell fate. Some studies show that human bone marrow MSC‐secreted ECM constructs induce osteogenesis (Bourgine et al., 2014a; Deng et al., 2016; Harvestine et al., 2016; Sutha et al., 2015; Zhou et al., 2016). However, the acquisition of human bone marrow MSCs (hBMSCs) is an invasive approach and the isolation is limited to the nutritional status and age of donors. For the clinical application of ECM constructs, hBMSC is not a best source of MSC. MSCs isolated from human umbilical cord (hUCMSCs), which is routinely discarded as clinical waste, has the similar capacities of cell proliferation, differentiation and immunosuppression to human bone marrow MSCs (Ennis, Gotherstrom, Le Blanc, & Davies, 2008; Ullah, Subbarao, & Rho, 2015). hUCMSC has been demonstrated to be a promising alternative source of MSC (Hou et al., 2009; Klontzas, Kenanidis, Heliotis, Tsiridis, & Mantalaris, 2015; Marupanthorn, Tantrawatpan, Kheolamai, Tantikanlayaporn, & Manochantr, 2017). However, the role and mechanism of the hUCMSC‐secreted ECM constructs (*hUCMSC‐ECM*) on bone formation remain unknown in vivo. The *hUCMSC‐ECM* has been demonstrated to retain a variety of cytokines secreted from hUCMSC, including insulin‐like growth factor binding protein 3 (IGFBP3) which is the most abundant cytokine deposited in the *hUCMSC‐ECM* (Deng et al., 2016). IGFBP3 belongs to the IGF binding protein family (IGFBPs) and is a major protein binding with circulating IGF‐I /II, which is the most abundant IGFBP in bone tissue (Baxter, 2013; Bhattarai, Lee, Lee, Park, & Yi, 2015; Firth & Baxter, 2002). Some studies have demonstrated that IGFBP‐3 controls cell apoptosis‐and survival‐related functions on cancer cells (Galluzzi et al., 2012; Johnson & Firth, 2014). In addition, IGFBP3 recruits a variety of resident cells toward injury sites, including hematopoietic stem cells/endothelial precursor cells and hepatic stellate cells (Chang et al., 2007; Kielczewski et al., 2009; Mannaerts et al., 2013). The role of IGFBP3 from *hUCMSC‐ECM* on the recruitment of MSC remains unclear. Recruitment of enough endogenous MSCs is the first stage in situ tissue regeneration (Vanden Berg‐Foels, 2014). If IGFBP3 is an MSC homing molecule, it is logical to assume that IGFBP3 might play an important role in the *hUCMSC‐ECM* recruiting endogenous MSCs to initiate bone formation.

In this study, we estimated bone formation of *hUCMSC‐ECM* in vivo using a 2‐mm severe combined immunodeficiency (SCID) mouse femur defect model compared to hUCMSC‐seed scaffold (*Vital*) and the scaffold substrate (*DBM*). Next, we analyzed the promigratory signaling pathway and relative molecule of homing cells recruited by *hUCMSC‐ECM* in vivo, and investigated the promigratory signaling pathway of *hUCMSC‐ECM* on hBMSCs in vitro. Finally, we demonstrated that *hUCMSC‐ECM* could recruit resident MSCs to initiate bone formation by IGFBP3 signaling pathway in vivo.

## 2 MATERIALS AND METHODS

### 2.1 Culture of cells and preparation of scaffolds

The protocols for isolating hUCMSCs and hBMSCs were as reported previously (Deng et al., 2016). MSCs were cultured in DMEM/F12 (HyClone, GE Healthcare life sciences, UT) with 10% fetal bovine serum (FBS; Gibco by life technologies corporation, NY), 0.1% penicillin, and streptomycin. The identification of MSCs was conducted on the basis of the criteria established by International Society for Cellular Therapy (ISCT) and assessed with using the human MSC Analysis Kit (562245, BD Biosciences, NJ) (Dominici et al., 2006; Squillaro, Peluso, & Galderisi, 2016). Passage 3–5 hUCMSCs were used for the preparation of scaffolds. Passage 3–5 hBMSCs were used for the cell migration experiments, qPCR, and Western blot.

Demineralized bone matrix (DBM) (10 × 5 × 5 mm) was prepared with ox cancellous bone as previously described (Hou et al., 2010). After pretreatment of DBM scaffolds in DMEM/F12 medium overnight, hUCMSCs were seeded onto DBMs at 1 × 10^6^ cells/scaffold to prepare the *Vital*. The *Vital* were further cultured in complete medium for 14 days, rinsed with PBS, flash frozen in liquid nitrogen for 10 min, thawed in a 37°C water bath with shaking at 60 rpm for 20 min, rinsed with PBS, frozen at −80°C overnight, and freeze‐dried to prepare the *hUCMSC‐ECM*. For the graft transplant to the SCID mice femur defect, DBM (3 × 3 × 3 mm, seeded with 4 × 10^4^ cells/scaffold), were used to prepare the *Vital* and the *hUCMSC‐ECM*.

To study the role of IGFBP3 from the *hUCMSC‐ECM* on cell migration, the *hUCMSC‐ECM* with an *Igfbp3* knock‐down model was used in this study. The short hairpin RNA transfection was conducted in hUCMSCs. hUCMSCs were cultured in DMEM/F12 medium containing Igfbp3 shRNA (lenti‐650004YY2502, OBIO technology co.LTD, Shanghai, China) for 24 hr, and then culture medium was changed into complete culture medium containing 2.5 pM puromycin amino nucleoside (Sigma, Saint Louis, MO) to eliminate the untransfected cells. The transfected cells were used to prepare the *shIgfbp3 hUCMSC‐ECM* (*shIgfbp3*) and a non‐silencing shRNA as a control (*shCtrl*).

### 2.2 Scanning electron microscopy (SEM) analysis

Scaffolds were fixed with glutaraldehyde, dehydrated in a gradient, and sealed with tert‐butyl alcohol. The scaffolds were then sprayed with gold and examined using a scanning electron microscope (S3400N II; Hitachi, Tokugawa, Japan) with an accelerating voltage of 15 kV and a working distance of 20–60 mm.

### 2.3 Cytokine quantification

Total proteins in DBM*, Vital*, and *hUCMSC‐ECM* were extracted with RIPA lysis buffer containing PMSF (Beyotime, Nanjing, Jiangsu, China), Cytokine levels were measured with ELISA kits (ELH‐IGFBP3‐001, ELH‐bFGF‐001; ELH‐OPG‐001; RayBiotech, Norcross, GA).

### 2.4 Immunofluorescent staining

For the in vitro assay, scaffolds were washed with PBS, blocked with 3% bovine serum albumin (BSA) for 30 min, and incubated overnight at 4°C with primary antibodies against IGFBP3 (1:50, rabbit anti‐human, Santa Cruz) in addition to 2U of Rhodamine Phalloidin (Biotium, Hayward, CA); DAPI was used to stain nuclei.

For the in vivo assay, the scaffolds, retrieved at 7 days in vivo, were embedded in optimal cutting temperature compound, and snap frozen at −20°C. Sections (8 µm thick) were incubated overnight at 4°C with primary antibodies against Sca‐1 (1:500, 7 H4 L3, Invitrogen, CA) and PDGFR‐α (1:500, Invitrogen). Secondary antibody labeled with Alexa Fluor 488 (1:100, donkey anti‐rabbit) or Cy3 (1:100, goat anti‐rat, ZSGB‐BIO, Beijing, China) were used as appropriate. DAPI was used to stain nuclei. The number of Sca‐1^+^ PDGFR‐α^+^ cells were counted and the percentage of Sca‐1^+^ PDGFR‐α^+^ cells to the total cells were determined Fluorescence images were acquired using a Two Photon Laser Scanning System (LSM 510 NLO, Zeiss, Oberkochen, Germany). A total of three images per animal distributed within the defect area, with 800× magnification, were analyzed.

### 2.5 Cell migration

The migration assay was carried out according to the methods published in our previous study (Deng et al., 2016). Briefly, 1.5 × 10^4^ hBMSCs in 100 µl of serum‐free culture medium were placed in the upper chambers of 24‐well Transwell plates (8 mm, Millipore, Darmstadt, Germany). The different concentrations of IGFBP3 or scaffolds (DBM, *hUCMSC‐ECM, shIgfbp3*, or *shCtrl*) were added in the lower wells with 1 ml of culture medium. For inhibition of the promigratory signaling pathway, hBMSCs were pretreated with 2 µM of SB505124 (for TβR I/II signaling pathway; Sellleckchem.cn, China), 500 nM BMS CCR2 22 (for CCR2 signaling pathway; Minneapolis, MN) for 1 hr prior to cell seeding. After 24 hr of incubation, the non‐migrated cells to the lower side of the filter were removed with cotton swabs, while the migrated cells were fixed with 4% paraformaldehyde and stained with crystal violet. The number of migrated cells was counted in three different fields under a microscope at 100× magnification. The data were averaged from three parallel experiments.

### 2.6 Quantitative real‐time polymerase chain reaction

For the in vitro experiment, the expression of receptor genes related to cell migration (PDGF‐Rα, PDGF‐Rβ, CCR4, IGF1R, TβR I, CCR2, and CXCR4) was examined by quantitative PCR. Briefly, for the in vitro experiment, hBMSCs were collected after 1, 2, and 3 days of incubation in culture media containing 25 ng/ml IGFBP3 (Peprotech, Rocky Hill, NJ), respectively. For the in vivo experiment, the expression of receptor genes related to cell migration (TβR I and CCR2) was examined. The tissue from scaffolds (DBM, *shCtrl*, and *shIgfbp3*) were collected at day 7 post implantation. Total RNA was extracted with TRIzol reagent (Invitrogen, Waltham, MA) and reverse transcribed with the PrimeScript™ RT reagent kit (Takara, Shimogyo‐ku, Kyoto, Japan) according to the manufacturer's instructions. Real‐time PCR was performed using 2 × SYBR Green PCR Master Mix Biosystems 7500). All the primer sequences (Sangon Biotech Co., Ltd., Shanghai, China) were designed using primer 5.0 software and these sequences are summarized in Supplemental Table S1.

### 2.7 RNA sequencing

RNA extraction, cDNA Library Construction, and RNA sequencing: Total RNA of retrieved scaffolds from bone defects on day 7 postimplantation, was extracted with TRIzol reagent (Invitrogen, Carlsbad, CA), according to the manufacturer's protocol. The RNA with RIN >8.0 were right for construction of a complementary DNA (cDNA) library. cDNA libraries were constructed for each pooled RNA sample using the VAHTSTM Total RNA‐seq (H/M/R) (Invitrogen) according to the manufacturer's instructions. The cDNA libraries were constructed for each pooled RNA sample using the NEBNext® Ultra™ Directional RNA Library Prep Kit (New England Biolabs, Ipswich, MA) for Illumina according to the manufacturer's instructions. The tagged cDNA libraries were pooled in equal ratio and used for 150 bp paired‐end sequencing in a single lane of the Illumina HiSeqXTen and sequencing with an Illumina HiSeqXTen system

#### 2.7.1 Dif‐gene‐finder

We applied DESeq algorithm (Anders & Huber, 2010) to filter the differentially expressed genes, after the significant analysis, *p*‐value and FDR analysis under the following criteria (Benjamini, Drai, Elmer, Kafkafi, & Golani, 2001), mRNA under the following criteria: (i) Fold Change >2 or <0.5 and (ii), FDR <0.05.

#### 2.7.2 Pathway analysis

Pathway analysis was used to find out the significant pathway of the differential genes according to KEGG database. We turn to the Fisher's exact test to select the significant pathway, and the threshold of significance was defined by *p*‐value and FDR.

### 2.8 Western blotting

To analyze the effects of IGFBP3 on the promigratory signaling pathway, hBMSCs were treated with culture medium containing 25 ng/ml IGFBP3 with or without pretreatment with 2 µM of SB505124 for inhibition of TβR I/II or p‐Smad 2/3, 500 nM BMS CCR2 22 for inhibition of CCR2. The hBMSCs were collected with or without IGFBP3 treatment for 6 hr, 12 hr, 1, 2, and 3 days, respectively. Total protein was extracted with 100 µl RIPA lysis buffer (P0013B, Beyotime, Jiangsu, China), subjected to SDS–PAGE, transferred on nitrocellulose membranes (Millipore, Billerica) and probed with specifics primary antibodies against p‐Smad 2/3 (Cell Signaling Technology), Smad 2/3(Santa Cruz Biotechnology, Texas), CCR2 (Santa Cruz Biotechnology) or TβR I/II (Santa Cruz Biotechnology), or GAPDH (Beyotime, Jiangsu, China) at 1: 500 dilution overnight at 4°C. Immunoreactive protein bands were visualized using ECL chemiluminescence detection plus a Western blot detection system (Bio‐Rad). The intensity ratio was the relative expression of p‐Smad2/3, Smad 2/3, TβR I, TβR II, and CCR2 normalized to GAPDH.

### 2.9 Animal surgical procedures and experimental design

All animal care and experimental protocols complied with the Animal Management Rule of the Ministry of Public Health, China (documentation 55, 2001). Eight‐week‐old SCID mice (weighing approximately 25–30 g, from the Animal Experiment Center of Southwest hospital of China) underwent a femoral osteotomy. The surgical procedure was performed as previously reported (Tsang et al., 2013; Xing et al., 2014). Briefly, SCID mice were anaesthetized and stabilized with fixation plates. A unilateral (or bilateral) 2‐mm segmental defect with removal of periosteum was created in each mouse. The different scaffolds were transplanted into the bone defects. The wounds were closed using standard surgical procedures. Mice were randomly assigned to five groups: the DBM group (*n* = 34), *Vital* group (*n* = 8), *ECM* group (*n* = 8), *shCtrl* group (*n* = 30), and *shlgfbp3* group (*n* = 30). To test the osteoinductive properties and colonization of host MSCs, scaffolds were retrieved at 7 days postoperatively and underwent immunofluorescent staining. At 3, 7, 10, and 14 days postoperatively, the recruited endogenous MSCs of the DBM, *shCtrl*, and *shlgfbp3* group were counted by flowcytometry. At 4 and 8 weeks postoperatively, the development of new bone in the defects was monitored by micro CT and the healing capacity of different treatments was further confirmed by histology assessment.

### 2.10 Flowcytometry

At 3, 7, 10, and 14 days postoperatively, the scaffolds were collected, cut into small pieces, and placed in a centrifuge tube. Cells were collected by digestion of defects with Tryple express (Gibco, life technologies, Denmark) and 0.1% type Ι collagenase (Solarbio Science & Technology Co., Ltd, China). Cells were labeled with the following antibodies: Rat anti‐Mouse CD45 (APC, clone 30‐F11, BD Medical Technology, NJ), Rat anti‐Mouse Sca‐1 (FITC, Clone D7, BD Medical Technology), and Rat anti‐Mouse PDGFR‐α (PE, clone APA5, BD Medical Technology). Data were acquired using a FACS Calibur flow cytometer with CellQuest Pro software (BD Biosciences) and analyzed with FlowJo (Tree Star, Inc., CA). The numbers of Sca‐1^+^ PDGFR‐α^+^ CD45^−^ cells in different groups were counted and the percentages of Sca‐1^+^ PDGFR‐α^+^ CD45^−^ cells in different groups were determined. The data shown are the mean from three independent experiments.

### 2.11 Micro‐CT

New bone formation at week 4 and 8 was evaluated with micro‐CT (Skyscan, Antwerp, Belgium). The regenerated femora with muscles removed in 4% paraformaldehyde were scanned with the following settings: voxel size 10.0 µm, voltage 65 kV, current A, exposure time 280 ms. The data were subsequently analyzed and imaged using CT Analyser software (version 1.16.1.0, Skyscan1272, Bruker Microct, Kontich, Belgium). 3‐D pictures were created with CTvox software (version 3.2.0r1294, Skyscan1272, Bruker Microct) (Seebach, Freischmidt, Holschbach, Fellenberg, & Richter, 2014). For all the regenerated bone within the defects, the elliptical region of interest (ROI) was 80 × 55 pixel, the number of slices from 264 to 1500 mg HA/cm^3^. For BMD estimation, BMD phantom has concentration of 0.25 g/cm^3^ CaHA. Relative bone volume per tissue volume(BV/TV), Trabecular number (Tb.N), and bone mineral density (BMD) of the regenerated bone within the defects were calculated using CTvox software (version 3.2.0r1294, Skyscan, Antwerp, Kontich, Belgium) (Das, Segar, Hughley, Bowers, & Botchwey, 2013).

### 2.12 Histology assessment

The femurs were retrieved. The muscle and soft tissue were stripped off. Next, the scaffolds or regenerated tissue were fixed in 4% buffered paraformaldehyde, decalcified in 50 mmethylenediaminetetraacetic acid, embedded in paraffin and sectioned at 4–6 mm thickness. The slides were used for H‐E staining.

### 2.13 Statistical analysis

One‐way ANOVA followed by Tukey's test was utilized to determine the statistical significance of the differences inimmunofluorescent staining and cell migration. Two‐way ANOVA followed by Sidak's multiple comparisons test was performed to determine the statistical significance of the real‐time PCR, WB, flow cytometry, and Micro CT data. EB‐seq was used for Dif‐Gene‐Finder and Fisher's exact test was used to select the significant pathway. The results are displayed as the mean stand ±standard deviation for *n* ≥3 scaffolds per group in all cases, unless otherwise indicated. For all the statistical tests, differences were considered to be significant if *p *< 0.05.

## 3 RESULTS

### 3.1 Osteoinductive property of the *hUCMSC‐ECM* was similar to the *Vital* and superior to scaffold substrate

**Figure 1:**
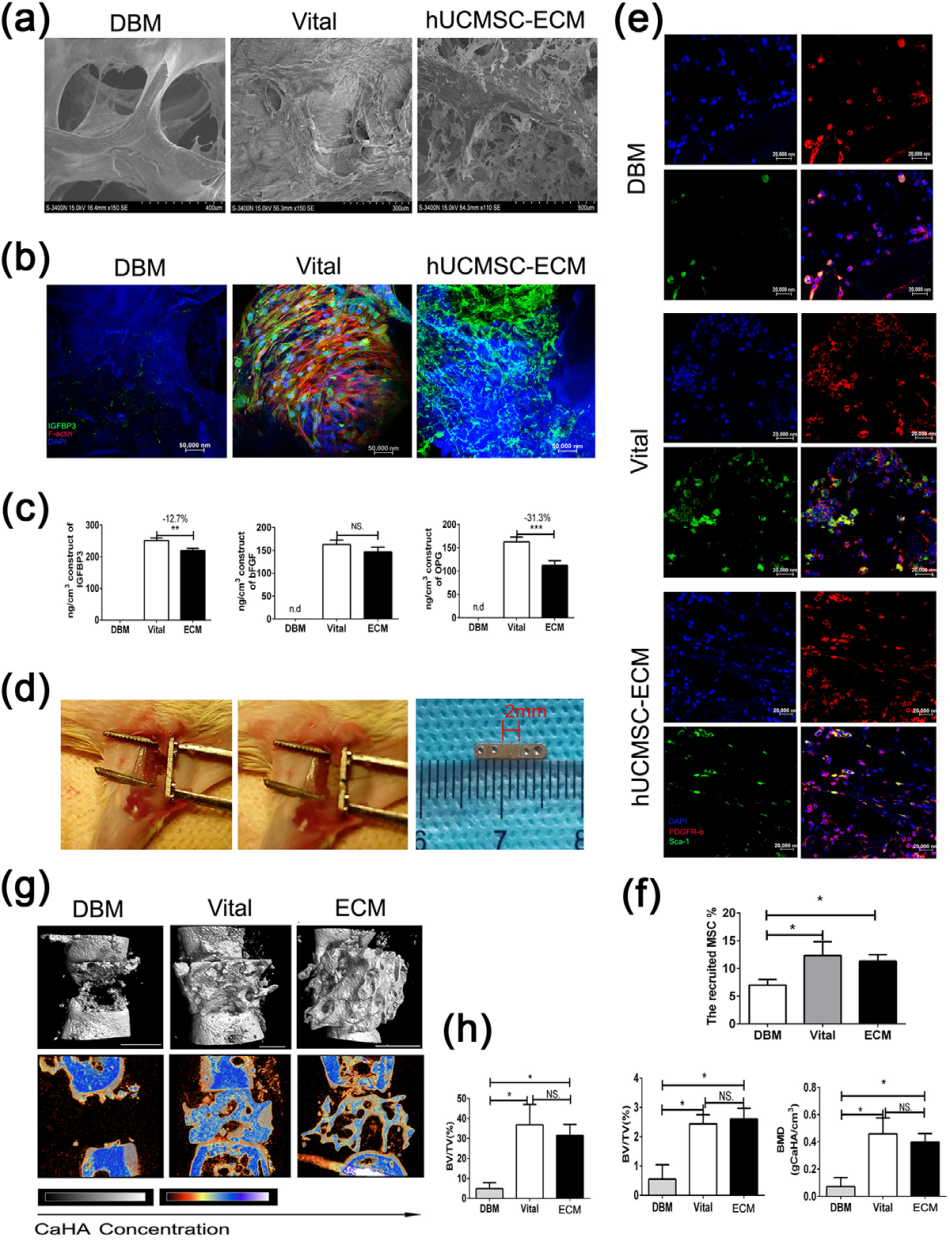
Surface morphological assay, IGFBP3 immunofluorescent images, host MSC recruitment, and micro. CT analysis of different matrix groups. (a) Surface morphological analysis of lyophilized DBM, Vital and hUCMSC‐ECM. (b) Immunofluorescent images of IGFBP3 in DBM, Vital, and hUCMSC‐ECM groups, respectively; red: F‐actin; green: IGFBP3; blue: DAPI; Scale bar: 50,000 µm. (c) The contents of IGFBP3, bFGF, and OPG in the total protein extract of DBM, Vital, and hUCMSC‐ECM. (d) Animal surgical procedure, the unilateral 2 mm segmental defects were created in each mouse. A 3 × 3 × 3 scaffolds were transplanted into the bone defects. (e) Representative immunofluorescent images of the host MSC in DBM, Vital, and hUCMSC‐ECM groups at day 7 postoperation; red: PDGFR‐α; green: Sca‐1; blue: DAPI; Scale bar: 20,000 µm. (f) Quantification of homing MSCs in (E). (g) 3D and 2D center‐sagittal view images of regenerated bone mass in the DBM, Vital, and hUCMSC‐ECM groups at 4 weeks postoperation Scale bar:1 cm. (h) BV/TV, Tb.N, and BMD of the regenerated bone in (G); *p < 0.05, **p < 0.01, ***p < 0.005

First, we tested whether the *hUCMSC‐ECM* was capable of inducing bone regeneration (Deng et al., 2016). The hUCMSCs isolated from umbilical cord Wharton's Jelly were identified and shown the characteristics of MSCs (Supplemental Figure S1). Primary hUCMSCs were seeded on DBM and cultured for 2 weeks in complete culture medium (DBM, *Vital*; Figures 1a and 1b). The *hUCMSC‐ECM* successfully displayed scaffolds surrounded by a protein‐rich surface (*hUCMSC‐ECM;* Figure 1a). The *hUCMSC‐ECM* was positively stained for IGFBP3, but not for cell components (F‐actin with red staining was not observable; Figure 1b). Quantitative assessments indicated that the loss of the principal cytokine amount was less than 1/3 amount of *Vital*, including IGFBP3 (−12.7%), bFGF (non significant difference), and OPG (−31.3%) (Figure 1c).

A large femur defect model of SCID mice was used to assess osteoinductivity of scaffolds (Figure 1d). The colonization of host cells was evident in the *Vital* and, to a lower extent, in the *hUCMSC‐ECM*, and DBM were colonized by a few resident cells (blue DAPI staining, Figure 1e). The homing MSCs (cells co‐labeling with green Sca‐1 staining and red PDGFR‐α staining) in the *Vital* and *hUCMSC‐ECM* groups were more than those in DBM group (*p* < 0.05 for the *Vital* or *hUCMSC‐ECM* groups vs. the DBM group, Figure 1f). Defects in the *Vital* group and the *hUCMSC‐ECM* group were extensively regenerated, resulting in interconnected mineralized bone trabeculae (Figure 1g). In contrast, the retrieved DBM maintained less bone formation and showed a clear defect gap (Figure 1g). Quantitative analysis of micro‐CT at 4 weeks post implantation showed BV/TV, Tb.N, and BMD of the regenerated bone in *hUCMSC‐ECM* were 10.2 ± 3.2‐, 6.9 ± 2.4‐, and 8.4 ± 3.4‐fold higher than those of DBM, respectively, which indicated that *hUCMSC‐ECM* remained superior to DBM (*p *< 0.05, Figure 1h). There was no difference between the *Vital* and *hUCMSC‐ECM* in BV/TV, Tb.N, and BMD, indicating that the *hUCMSC‐ECM* had the same bone formation capacity as the *Vital* (Figure 1h).

### 3.2 The *hUCMSC‐ECM* upregualted the pro‐migratory gene expression and hBMSC migration

**Figure 2:**
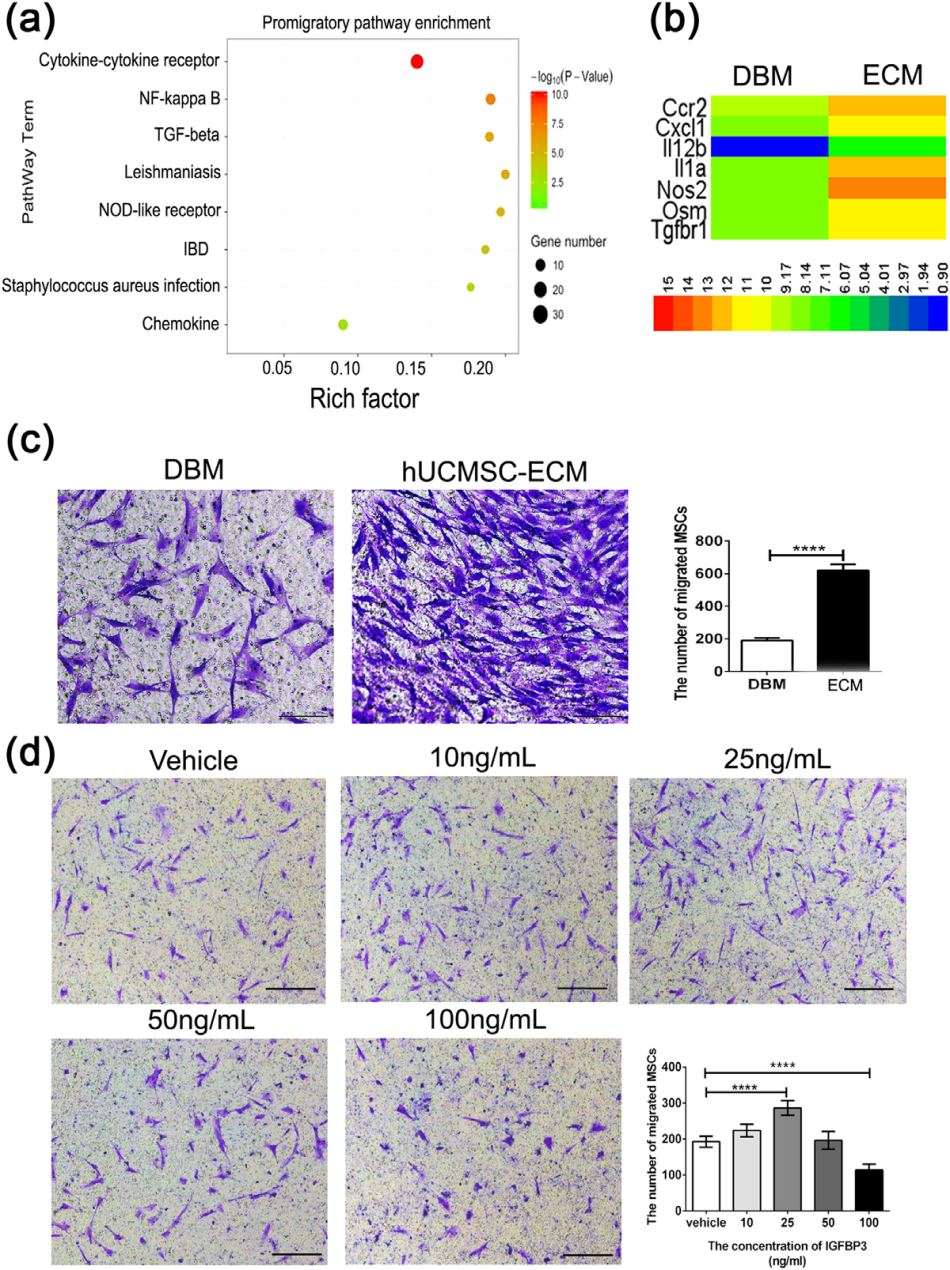
Transcriptome analyses of the homing cells collected from scaffolds and transwells assay for scaffolds‐induced hBMSC migration and IGFBP3‐induced hBMSC migration. (a) The enrichment analysis of promigratory pathways of the recruited cells on day 7 postimplantation. (b) Heatmap showing the seven genes of hUCMSC‐ECM group significantly upregulated fivefold than those of DBM group on day 7 postimplantation. (c) Representative light photomicrographs of migrated hBMSC induced by scaffolds after 24‐hr incubation. (d) Representative light photomicrographs of migrated hBMSC induced by IGFBP3 after 24‐hr incubation. The migrated cells were stained purple with crystal violet; Scale bar: 100 µm. ****p < 0.001

Besides osteoinductive capacity, the osteoinductive mechanism of the *hUCMSC‐ECM* has attracted most scientific interest. The osteoinductivity of ECM has been considered relying on a mixture of factors accumulated at doses within physiological ranges (Bourgine et al., 2014a). This is not a specific mechanism, but just a popular recognition. Recent some successful proof‐of‐concept studies confirm that the first stage of in situ tissue regeneration must result in recruitment of endogenous MSCs and cells (Vanden Berg‐Foels, 2014). Figure 1e had shown that more host cells were colonized in the *hUCMSC‐ECM* than DBM (blue DAPI staining, Figure 1e). Furthermore, transcriptome analysis of promigratory genes significantly upregulated in homing cells collected from the *hUCMSC‐ECM* was conducted compared to DBM. The enrichment analysis of pathways demonstrated eight pathways were significant (*p* < 0.01) and the dominant pathway related to cell migration was the cytokine–cytokine receptor in interaction signaling pathway, enriching more than 30 cytokine receptors which included many CCRs and CXCRs cytokine receptors (−log10=10.04p) (Figure 2a). Besides, NF‐κβ signaling pathway (−log10=7.51p), NOD‐like receptor signaling pathway (−log10=5.11p), and TGFβ signaling pathway (−log10=4.50p), paid an important role on cell homing (Figure 2a). Among the upregulated 89 genes being coincident in the significant promigratory pathways, seven differentially‐expressed genes of ECM group upregulated more than fivefolds than that of DBM group, shown in the heatmap (Figure 2b and supplemental Figure S2). The seven genes included CXCR1, an important regulator of vascular endothelial cell migration, TGFβ1, and CCR2, the pro‐migratory receptors of MSCs (Ponte et al., 2007; Wan et al., 2012; Zeng, Shen, Huang, Liu, & Liu, 2012).

Stem cell migration is the first step of cell recruitment (Vanden Berg‐Foels, 2014). Prompted by the immunofluorescence results that more host MSCs were colonized in *hUCMSC‐ECM* on 3–14 days post implantation, we investigated whether *hUCMSC‐ECM* played a positive role in MSCs migration in vitro. After 10‐hr stimulation, the number of migrated hBMSC in *hUCMSC‐ECM* group was 3.3 ± 0.1‐fold higher than that of DBM groups (*p *< 0.001; Figure 2c).

### 3.3 IGFBP3 promoted the migration of hBMSCs

The mode of recruitment is directional migration in response to a gradient of soluble chemoattractants, including growth factors (GFs) and chemokines (Lairdvon, von Andrian, & Wagers, 2008). In a previous study, quantitative cytokine array analysis showed that the amount of IGFBP3, the principal component in *hUCMSC‐ECM*, accounted for 23% of the total amount of 56 cytokines tested (Deng et al., 2016). Furthermore, IGFBP3 could stimulate the migration of hematopoietic stem cells (Kielczewski et al., 2009; Ponte et al., 2007). We therefore evaluated the role of IGFBP3 in hBMSC migration. A Transwell assay showed that hBMSCs migration was correlated with the concentration of IGFBP3. When its concentration was not higher than 25 ng/ml, IGFBP3 promoted hBMSCs migration in a concentration‐dependent manner. At 25 ng/ml of IGFBP3, the number of migrated cells reached a peak and was nearly 1.5 ± 0.1‐fold higher than in the vehicle control. In contrast, at the concentration of IGFBP3 higher than 25 ng/ml, IGFBP3 inhibited cell migration (*p* < 0.001, Figure 2d).

### 3.4 IGFBP3‐induced hBMSC migration required the activation of the TGFβ and CCR2 signaling pathways

The promigratory activity of IGFBP3 on hBMSCs has rarely been reported and the acting signaling pathway of IGFBP3 is of interest. In contrast to HSCs, whose migration is induced mainly by SDF‐1, the migratory capacity of MSCs could be affected by various chemotactic factors and receptors (Ponte et al., 2007). López Ponte et al. (2007) reported that some membrane receptors were particularly relevant for adult bone marrow MSC homing, such as PDGF‐Rβ, PDGF‐Rα, IGF‐1R, CCR2, CCR4, and CXCR4. Furthermore, the TGFβ signaling pathway was demonstrated to be associated with IGFBP3‐induced migration of hepatic stellate cells (Mannaerts et al., 2013). Thus, we evaluated the roles of IGFBP3 on the above mentioned receptors.

**Figure 3:**
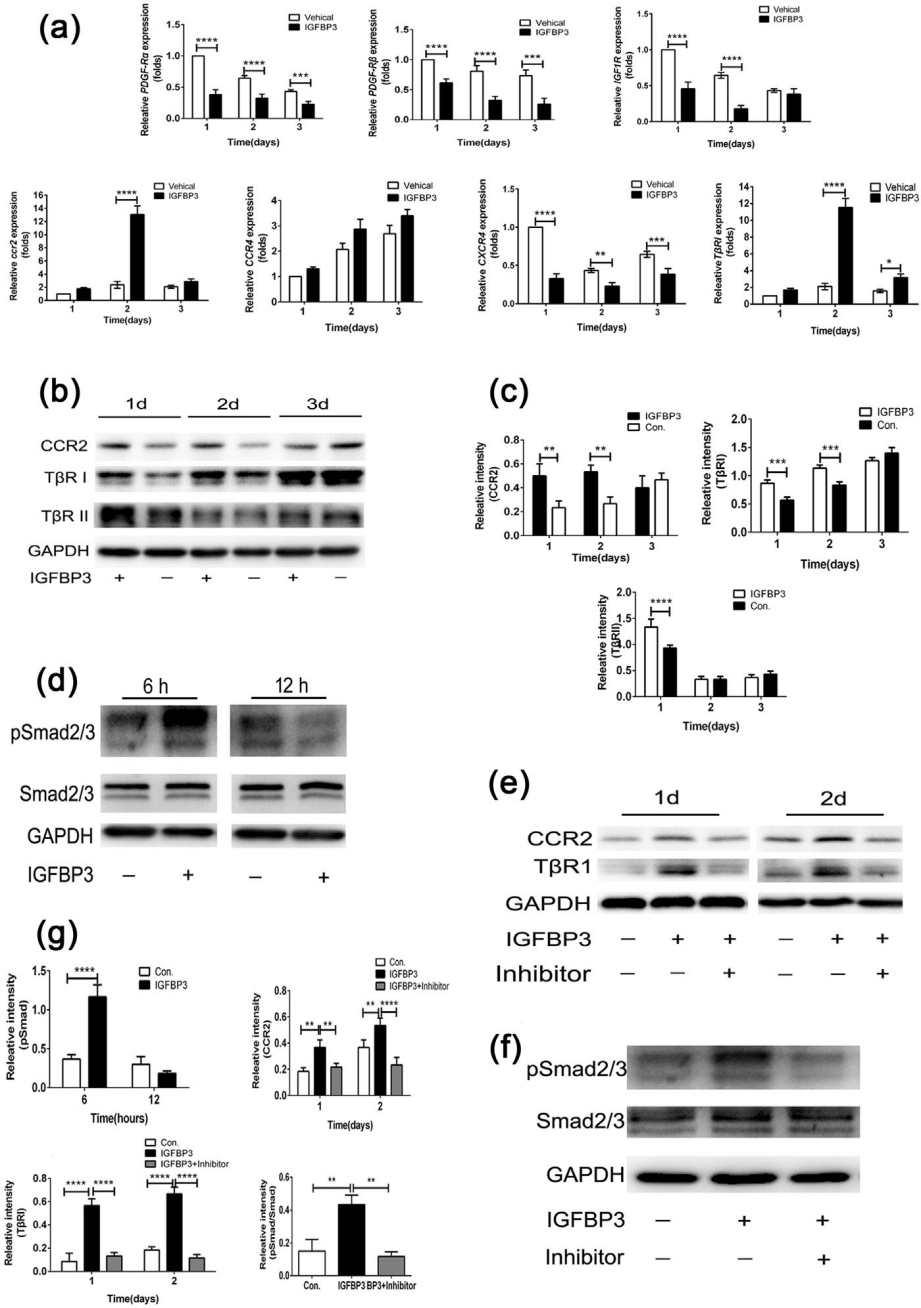
The expressions of gene and protein of hBMSC treated with or without IGFBP3 at different times. (a) The relative expressions of PDGF‐Rβ, PDGF‐Rα, IGF‐1R, CCR2, CCR4, CXCR4, and TβRI of hBMSC in vehicle group and IGFBP3 group were analyzed by qRT‐PCR at 1, 2, and 3 days. (b) The expressions of CCR2, TβRI, and TβRII of hBMSC induced by IGFBP3 were assessed by Western blot at 1, 2, and 3 days. (c) The relative intensity of CCR2, TβRI, and TβRII normalized to GAPDH at 1, 2, and 3 days. (d) The expressions of pSmad2/3 and Smad2/3 were induced by IGFBP3 as assessed by Western blot at 6 and 12‐hr. (e) The expressions of CCR2, TβRI, and TβRII in hBMSC stimulated by IGFBP3 or primed with inhibitors were assessed by Western blot at 1 and 2 days. (f) The expressions of pSmad2/3 and Smad2/3 in hBMSC stimulated by IGFBP3 or primed with inhibitors were assessed by Western blot at 6‐hr. (g) The relative intensity of CCR2, TβRI, TβRII, pSmad2/3, and Smad2/3 in hBMSC stimulated by IGFBP3 or primed with inhibitors were assessed by Western blot at different times; 2 µM of SB505124 for inhibition of TβR I/II or p‐Smad 2/3, 500 nM BMS CCR2 22 for inhibition of CCR2; **p < 0.01, ***p < 0.005, ****p < 0.001

qPCR results showed that the relative expression of PDGF‐Rβ, PDGF‐Rα, IGF‐1R, and CXCR4 in the IGFBP3 group decreased heavily compared to those of the vehicle group (*p* < 0.001; Figure 3a). Meanwhile, the expressions of CCR2 and TβRI in the IGFBP3 group were nearly 5.6 ± 0.7‐ and 5.5 ± 0.5‐fold higher than those in the vehicle group, respectively, at day 2 of stimulation (*p* < 0.001; Figure 3a). There was no difference in the expression of CCR4 between the IGFBP3 and vehicle groups (Figure 3a). The qPCR results indicated that IGFBP3 improved the activation of CCR2 and TβRI. Then, Western bloting was used to estimate whether IGFBP3 stimulation would affect the proteins of the CCR2 and TβRI pathways. At 1 day of stimulation, the relative expressions of CCR2, TβRI, and TβRII in the IGFBP3 group were nearly 2.2 ± 0.3‐, 1.5 ± 0.1‐, and 1.4 ± 0.2‐fold higher than those in vehicle group, respectively, (*p* < 0.01 for CCR2; *p* < 0.005 for TβRI and TβRII, Figures 3b and 3c). The results indicated that CCR2 and TβRI/II were activated by IGFBP3 stimulation. After 6‐hr stimulation, the phosphorylation level of Smad 2/3 of the IGFBP3 group was 3.3 ± 0.6‐fold higher than that of the vehicle group, which verified the speculation that IGFBP3 could activate CCR2 and TβRI/II pathways (*p* < 0.001; Figures 3b–d and 3g).

**Figure 4:**
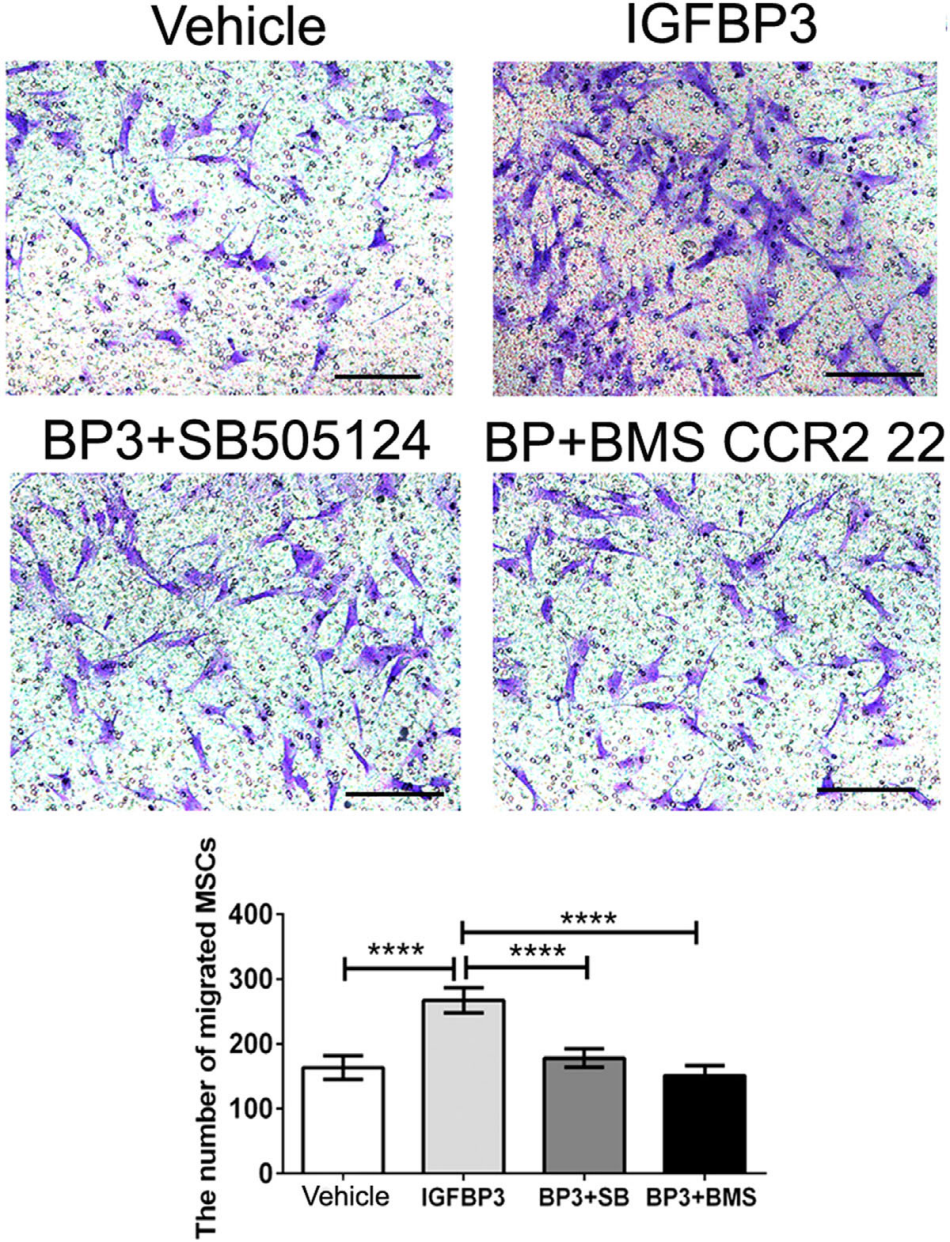
Transwells assay for MSC migration was induced by IGFBP3 with or without the pretreatment of inhibitors. The migrated cells were stained purple with crystal violet. Scale bar: 100 µm; ****p < 0.001

To demonstrate whether IGFBP3 could induce hBMSC migration via the TβRI/II and CCR2 signaling pathways, the two pathways were blocked with chemical inhibitors (SB505124 for the TβR I/II signaling pathway, BMS CCR2 22 for the CCR2 signaling pathway). The expressions of TβRI/II and CCR2, in combination with cell migration were evaluated. After pretreatment with inhibitors, the IGFBP3‐induced relative expressions of CCR2, TβRI and pSmad 2/3 decreased by 56.7 ± 5.6%, 82.1 ± 6.2%, and 73.3 ± 2.9%, relative to the vehicle group, respectively (*p* < 0.005 for CCR2 and *p* < 0.001 for TβRI and pSmad 2/3; Figure 3e–g). Accordingly, the number of IGFBP3‐induced migrated cells decreased by 33.2 ± 6.3% and 43.3 ± 5.5% for the inhibition of the TβRI/II pathway and CCR2 pathway, respectively (*p *< 0.001, Figure 4).

### 3.5 *hUCMSC‐ECM* induced the chemotaxis of hBMSCs by the IGFBP3 signaling pathway

**Figure 5:**
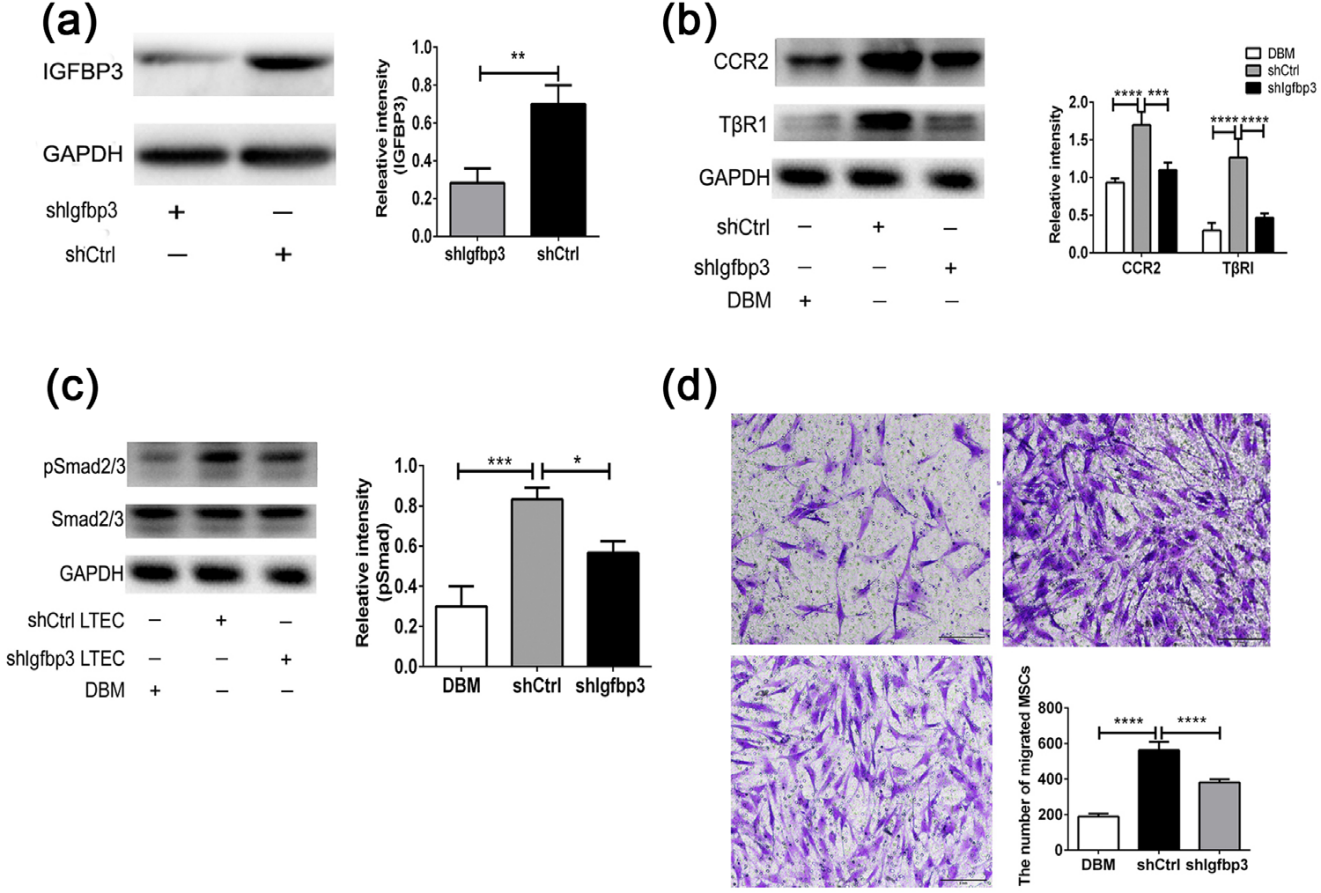
*hUCMSC‐ECM*‐induced migration of hBMSC by IGFBP3 signaling pathway. (a) IGFBP3 expression in the protein extract from *hUCMSC‐ECM* in which the seeding cells were transfected with shIgfbp3 as assessed by Western blot. (b) The expressions of CCR2 and TβRI of hBMSC induced by shIgfbp3, and shCtrl were assessed by Western blot. (c) The expressions of pSmad2/3 and Smad2/3 of hBMSC induced by shIgfbp3 and shCtrl were assessed by Western blot. (d) Transwells assay for MSC migration was induced by scaffolds; the migrated cells were stained purple with crystal violet. Scale bar: 100 µm; *p < 0.05, **p < 0.01, ***p < 0.005, ****p < 0.001

To investigate the roles of IGFBP3 on the *hUCMSC‐ECM*‐induced migration of hBMSCs, we prepared the *hUCMSC‐ECM* with a knock down of Igfbp3 and observed whether the migratory ability of hBMSC was inhibited when IGFBP3 signaling was blocked. After 14 days of culture, the relative expression of IGFBP3 in the *shIgfbp3* group was 40.2 ± 8.6% of that in the *shCtrl* group, suggesting a significant decrease of IGFBP3 expression in the *shIgfbp3* group (*p* < 0.01; Figure 5a). The relative expressions of CCR2, TβRI, and pSmad2/3 in the *shCtrl* group increased 83.3 ± 12.3%, 341.6 ± 29.0%, and 205.6 ± 14.3% compared to those of the DBM group, respectively. However, those in the *shIgfbp3* group decreased 35.5 ± 3.5%, 61.6 ± 10.7%, and 31.5 ± 8.2% compared to those of the *shCtrl* group, respectively (Figures 5b and 5c).This result suggested that the knock down of IGFBP3 in the *shIgfbp3* blocked the activation of the CCR2 and TβRI/II signaling pathways. Accordingly, Transwell results showed that inhibition of the IGFBP3 signaling pathway also suppressed the migratory ability of hBMSCs. Compared to the DBM group, the *shCtrl* enhanced 196.9 ± 13.5% of the migrated cell amount, while the *shIgfbp3* inhibited 47.1 ± 9.2% of the migrated hBMSC amount (*p* < 0.001, Figure 5d).

### 3.6 IGFBP3 deposited in the *hUCMSC‐ECM* recruited endogenous MSCs to initiate bone formation

**Figure 6:**
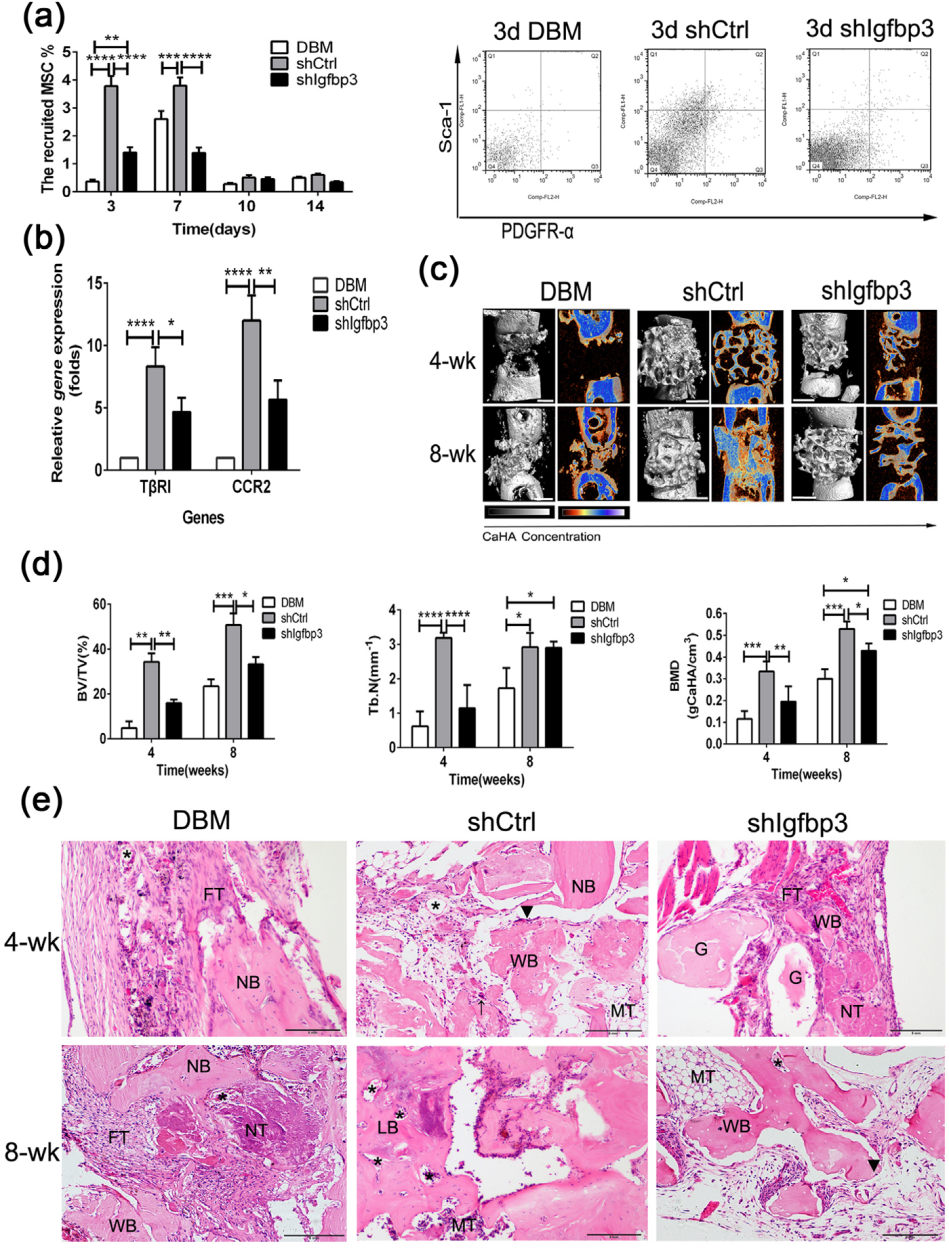
Role of IGFBP3 on hUCMSC‐ECM‐induced recruitment host MSCs toward bone regeneration. (a) The percentage of host MSC recruited on scaffolds was counted by flowcytometry at 3, 7, 10, and 14 days. (b) qPCR analysis for TβRI and CCR2 expressions. (c) 3D and 2D center‐sagittal view images of regenerated bone mass in the DBM, shIgfbp3, and shCtrl groups at 4 and 8 weeks postimplantation Scale bar:1 cm. (d) BV/TV, Tb.N, and BMD of the regenerated bone in (c). (d) Histological assessment of regenerated tissue in the DBM, shCtrl, and shIgfbp3 groups at 4 and 8 weeks postimplantation; G, graft; NB, native bone; WB, woven bone; LB, lamellar bone; FT, fibrous tissue; MT, medullary tissue; NT, necrotic tissue; (▴), osteoblast; (*), new vessels; (↑), osteoclast; Scale bar: 5 mm; *p < 0.05, **p < 0.01, ****p < 0.001

To assess whether IGFBP3 from the *hUCMSC‐ECM* could promote the recruitment host MSCs, DBM, the *shCtrl*, and the *shIgfbp3* were implanted into femur defects of SCID mice. Flowcytometry was used to quantify the homing of MSCs. At 3, 7, 10, and 14 days post implantation, MSCs collected from scaffolds were stained with Mouse CD45, Sca‐1and PDGFR‐α (Houlihan et al., 2012). The settings of these gates refer to the study of Houlihan et al. (2012) (Supplemental Figure S3). Representative Sca‐1 versus PDGFR‐α plots were gated on CD45^−^ cells in DBM, shCtrl, and shIgfbp3 groups in 3 days post‐implantation (Figure 6a). The percentage of recruited MSCs of the *shCtrl* reached a peak value at 3 days (3.8 ± 0.6% of the total account of cell number), and as well as the *shIgfbp3*, to a lower extent (1.4 ± 0.3%), whereas DBM obtained few MSCs (0.4 ± 0.1%) and peaked at 7 days (2.6 ± 0.5%). Collectively, the *shCtrl* recruited more host MSCs than DBM did at day 3 (10.6 ± 1.7‐fold more than DBM) and the knock down of IGFBP3 led a remarkable decrease of MSCs (−62.9 ± 3.8%; *p* < 0.001, Figure 6a). The data indicated the successful recruitment of the host MSCs by the *shCtrl*, but not by DBM or the *shIgfbp3*, which is a prerequisite for bone regeneration. Similar to the changes of different scaffolds‐induced endogenous MSC homing, the expressions of TβRI, and CCR2 were increased by *shCtrl* administration by 10.7 ± 2.5‐ and 7.3 ± 1.5‐folds, and followed by the inhibition of the *shIgfbp3* by 51.5 ± 6.5% and 44.3 ± 5.2%, respectively (DBM vs. *shCtrl* for *p* < 0.001, *shCtrl* vs. *shIgfbp3* for *p* < 0.01; Figure 6b).

The presence of new bone regeneration was used to estimate whether ECM‐induced host homing could initiate bone formation. Remarkably, the *shCtrl*, in strong contrast to DBM and the *shIgfbp3*, gave rise to bone structures at 4 weeks post implantation (Figure 6c). After segmentation of micro‐computerized tomography images, the amount of mineralized tissue was quantified. BV/TV, Tb.N, and BMD of the *shCtrl* group were highest, followed by the *shIgfbp3* ones, whereas those of DBM group were the least (*p* < 0.01 for the *shCtrl* vs. the *shIgfbp3* in BV/TV and BMD; *p* < 0.001 for the *shCtrl* vs. the *shIgfbp3* in Tb.N; Figure 6d). The BV/TV, Tb.N, and BMD of the *shIgfpb3* specimens were approximately 48.3%, 35.5% and 60.4% of the *shCtrl* ones at week 4, respectively. The results that the *shIgfpb3* resulted in inferior bone regeneration which might be attributed to the fact that the *shIgfbp3* recruited less homing MSCs, while the IGFBP3 signaling pathway played a significant role in the *shCtrl*‐induced osteogenesis (Figure 6d). H‐E histology showed that defects of DBM were filled by a layer of fibrous connective tissue, whereas woven bone filled in the defects of the *shCtrl* and a little new osteoid tissue was observed in the *shIgfbp3* specimens at 4 weeks post implantation. After 8 weeks, the *shCtrl* specimens had a bony bridging of the defects with bone marrow tissue, while the *shIgfbp3* specimens still were filled with cancellous bone and DBM had much less bone tissue (Figure 6e).

## 4 DISCUSSION

The present study confirmed the unreported capacity of *hUCMSC‐ECM* to induce bone regeneration in vivo. The *hUCMSC‐ECM* exhibited an osteoinductive nature similar to living *hUCMSC*‐seed scaffolds and superior to unmodified scaffolds. Second, *hUCMSC‐ECM* was confirmed to activate promigratory signaling pathway, upregulate promigratory gene expression and improve hBMSC migration. Third, we further showed that IGFBP3, the most abundant cytokine deposited in *hUCMSC‐ECM*, could enhance MSC migration via the TGFβ and CCR2 signaling pathways. Finally, we demonstrated that the *hUCMSC‐ECM* recruited endogenous MSCs to initiate bone formation by the IGFBP3 signaling pathway in vivo.

From the recent perspective of MSCs as an “injury drugstore,” the trophic effect of MSCs is over their direct participation to the tissue formation (Caplan & Correa, 2011; Sutherland, Converse, Hopkins, & Detamore, 2015). The trophic effect of MSCs is derived from the MSC secretome appropriately bound to the ECM. As a reservoir of many biochemical and mechanical signals, ECM components are at the center of this complex interplay and have a vital role in regulating physiological processes. Heloïse Ragelle et al. (2017) reported that ECMs secreted from different seeding cells displayed distinct features in instructing cell behaviors. Ivan Martin et al. documented that hBMSC‐secreted ECM had high levels of BMP2, VEGF, and OPG (Bourgine et al., 2014b). In our previous study, IGFBP3, bFGF, and OPG deposited in *hUCMSC‐ECM* were the three most abundant cytokines among 56 cytokines tested (Deng et al., 2016). In the present study, the three cytokines, which have been reported to be involved in cell migration, proliferation, angiogenesis, and bone formation, accumulated more than 100 ng/cm^3^ in the *hUCMSC‐ECM* (Figure 1c) (Baxter, 2013; Bhattarai et al., 2015; Lee, Lee, Cho, Kim, & Shin, 2015; Wang, Huang, Pan, Jiang, & Liu, 2010). The loss of these cytokine contents in the *hUCMSC‐ECM* was less than 30% compared to the living cells did. Thus, the microtomographic analysis showed the regenerated bone induced by the *hUCMSC‐ECM* was not significantly different from the *Vital* ones (Figure 1g).

MSC are recruited to locations in adult tissues mainly by migration through the vascular network (Vanden Berg‐Foels, 2014). The mode of recruitment used in tissue regeneration is chemotaxis, which is directional migrationin response to a release of chemoattractants including GFs and chemokines (Vanden Berg‐Foels, 2014). Although IGFBP3 is reported to recruit endothelial precursor cells and hepatic stellate cells, the role of IGFBP3 on MSC homing is still unclear (Kielczewski et al., 2009; Mannaerts et al., 2013). In the present study, Transwell data suggested that exogenous IGFBP3 remarkably improved hBMSC migration in a dose‐dependent manner (Figure 2b). The amount of IGFBP3 deposited in the *hUCMSC‐ECM* was as high as 219.5 ± 7.7 ng/cm3, enhanced migrated hBMSCs approximately twofold, and recruited 10‐fold more endogenous MSCs. The knock down of *Igfbp3* in the *hUCMSC‐ECM* down‐regulated approximately 60% expression of IGFBP3, reduced the number of migrated hBMSCs by migrated hBMSCs, and inhibited nearly 60% of MSC homing and bone regeneration capacity (Figures 5a and 5d; 6a and 6c). These data showed that IGFPB3 played a decisive effect on *hUCMSC‐ECM* recruiting MSCs to participate in bone formation.

**Figure 7:**
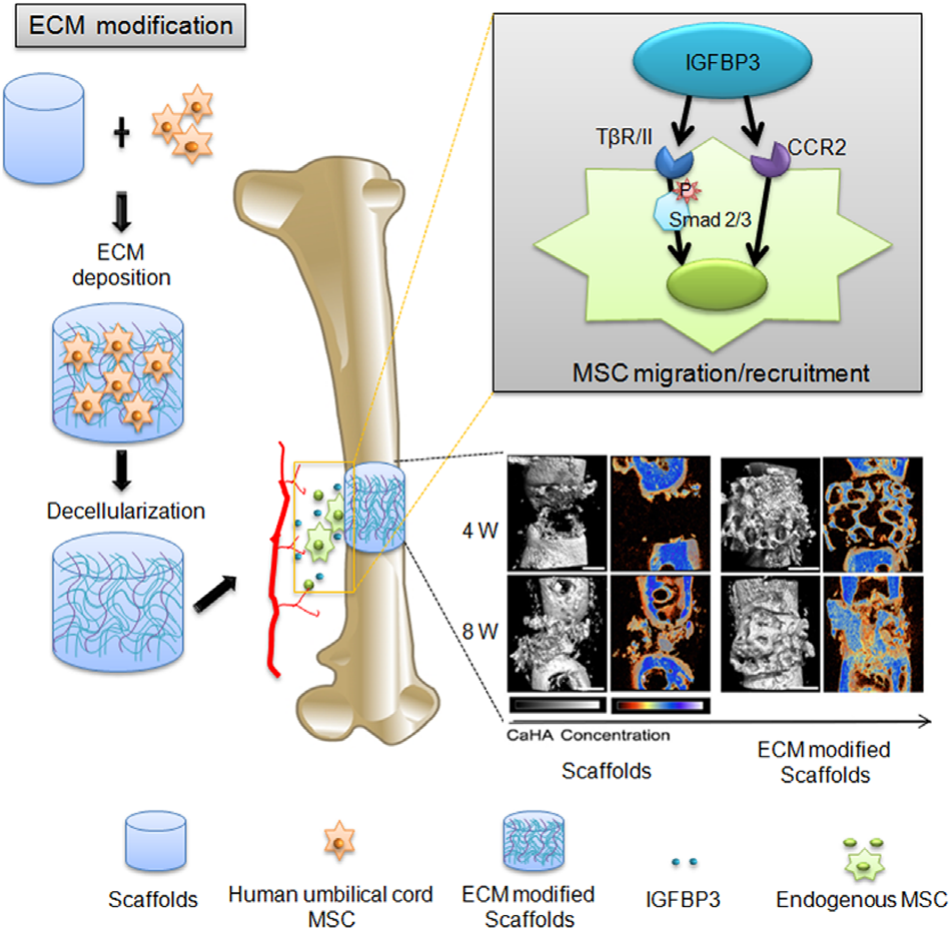
Scheme illustrates the mechanism of hUCMSC‐ECM recruiting endogenous MSC to initiate bone formation

In the previous study, the recruitment of hepatic stellate cell could be modulated by IGFBP3 through an interaction with TGFβ/Smad signaling (Mannaerts et al., 2013). Our further study of IGFBP3 on the promigratory signaling pathway was conducted by qPCR, Western blot and Transwell assay. IGFBP3 improved hBMSCs migration through TGFβ/Smad signaling pathways, which is consistent with the reported TGFβ‐dependent mechanism. Of note, IGFBP3‐induced hBMSC migration coincided with the activation of CCR2 and CCR2 inhibitors completely blocked the IGFBP3‐induced MSC migration, indicating that IGFBP3 promoted hBMSC migration through a CCR2‐dependent mechanism (Figures 3 and 4). On the other hand, genome‐wide association and functional studies have shown that IGFBP3 overexpression induced cartilage catabolism and osteogenic differentiation in hip osteoarthritis (Evans et al., 2015). Transcriptome analysis of homing cells showed that the *hUCMSC‐ECM* significantly increased the expressions of 7 promigratory genes, including TβRI and CCR2 in vivo (Figure 2a). While knock‐down of IGFBP3 of the *hUCMSC‐ECM* decreased the expression of TβRI and CCR2 in vivo, accompanied by the down‐regulation of endogenous MSC homing (Figure 6b). These data confirmed that IGFBP3 deposited from *hUCMSC‐ECM* can recruit MSCs by TβRI and CCR2 signaling pathway. A few studies demonstrated that IGFBP3 could improve osteogenesis through up‐regulation of p‐ERK signaling and down‐regulation of p‐JNK signaling, and decrease osteoclastogenesis through inhibition of RANKL signaling (Bhattarai et al., 2015). From a novel perspective, our data suggested that IGFBP3 induced bone formation through the increase of MSC homing in the TGFβ‐ and CCR2‐dependent mechanisms (Figure 7).

In the present study, we demonstrated that the surface modification of scaffolds with *hUCMSC‐ECM* could acquire an osteoinductive nature, similar to that of living cell‐seeded material and superior to the scaffold substrate. We presented a novel mechanism of IGFBP3 signaling in the migration of hBMSCs. Lastly, this study highlights the fact that the enhanced therapeutic potential of *hUCMSC‐ECM* by improving endogenous MSC homing in an IGFBP3‐dependent manner.

## Supporting information

Additional Supporting Information may be found online in the supporting information tab for this article.

**Figure S1**. Identification of hUCMSCs. A: Representative phase images of adherent P4 MSCs.
**Figure S2**. Heat map constructed by promigratory 89 genes derived from the significant pathways.
**Figure S3**. Endogenous MSC kinetics postimplantation.
**Table S1**. Gene primers for Real time PCR.
