# Supplementary material for: IGFBP3 deposited in the human umbilical cord mesenchymal stem cell‐secreted extracellular matrix promotes bone formation
Source: J Cell Physiol. 2018 Mar 1;233(8):5792–804. doi: 10.1002/jcp.26342 (PMC6220941; doi:10.1002/jcp.26342)
Supplement: Supplementary file 1 — Figure S1. Identification of hUCMSCs. A: Representative phase images of adherent P4 MSCs. Figure S2. Heat map constructed by promigratory 89 genes derived from the significant pathways. Figure S3. Endogenous MSC kinetics postimplantation. Table S1. Gene primers for Real time PCR. [file JCP-233-5792-s001.pdf]

Supplementary materials for review

IGFBP3 deposited in the human umbilical cord mesenchymal stem cell-secreted extracellular matrix promotes bone formation

Moyuan Deng [a*], Keyu Luo [a], Tianyong Hou [a], Fei Luo[a], Zhao Xie [a], Zehua Zhang[a], Aijun Yang [a], Bo Yu[a], Shaoxuan Yi [a], Shiwu Dong [a, b*], Jianzhong Xu [a*]

a National and Regional United Engineering Lab of Tissue Engineering, Department of Orthopaedics, Southwest Hospital, the Third Military Medical University, Chongqing, China

b Department of Biomedical Materials Science, College of Biomedical Engineering, Third Military Medical University, Chongqing, China

* Correspondence: Moyuan Deng (T: +86-023-68765286; F: +86-023-68765286, E-mail: betty.deng@126.com);Shiwu Dong (T: +86-023-68771270, F: +86-023-68771270, E-mail: dongshiwu@163.com); Jianzhong Xu (T: +86-023-68754164, F: +86-023-68773003, E-mail: jianzhong_xu@126.com)

Supplemental Table 1 Gene primers for Real time PCR

Gene	GenBank ID	Sequence	
TβR I	NM_001130916.2	Forward	GAGCCGCTGCCCATCGTGTAC
		Reverse	GGCGCCCAATGACACAGAGATC
CCR2	NM_001123041.2	Forward	TCGGCCTGAGTAACTGTGAAAGC
		Reverse	CTCCCCAACGAAGGCATAGATG
CXCR4	NM_001008540.2	Forward	TGCCCTCCTGCTGACTATTCC
		Reverse	AGGATAAGGCCAACCATGATG
PDGF-Rα	NM_001347827.1	Forward	TGAAGGCAGGCACATTTACATCTA
		Reverse	TACAGGAGTCTCGGGATCAGTTG
PDGF-Rβ	NM_002609.3	Forward	GGGGACAGGGAGGTGGATT
		Reverse	ATTCCCGATCACAATGCACA
CCR4	NM_005508.4	Forward	CAGCTCCCTGGAAATCAACATTC
		Reverse	CAGTCTTGGCAGAGCACAAAAAGG
IGF1R	NM_000875.4	Forward	ACCCGGAGTACTTCAGCGC
		Reverse	CACAGAAGCTTCGTTGAGAA
GAPDH	NM_001289746.1	Forward	CTTCAACAGCGACACCCACT
		Reverse	GTGGTCCAGGGGTCTTACTC
CCR2	NM_009915.2	Forward	GGAGAA AAGCCA ACT CCT TC
		Reverse	AGG CAG TTGCAA AGG TACTG

TβR I	NM_001312868.1	Forward	TGTGTCGAAAGCATGAAGGA
		Reverse	GGTCCCAGCACTCAGTCAAC
GAPDH	NM_001289726.1	Forward	TCAAGAAGGTGGGTGAAGCAGG
		Reverse	TATTATGGGGGTCTGGGATGG

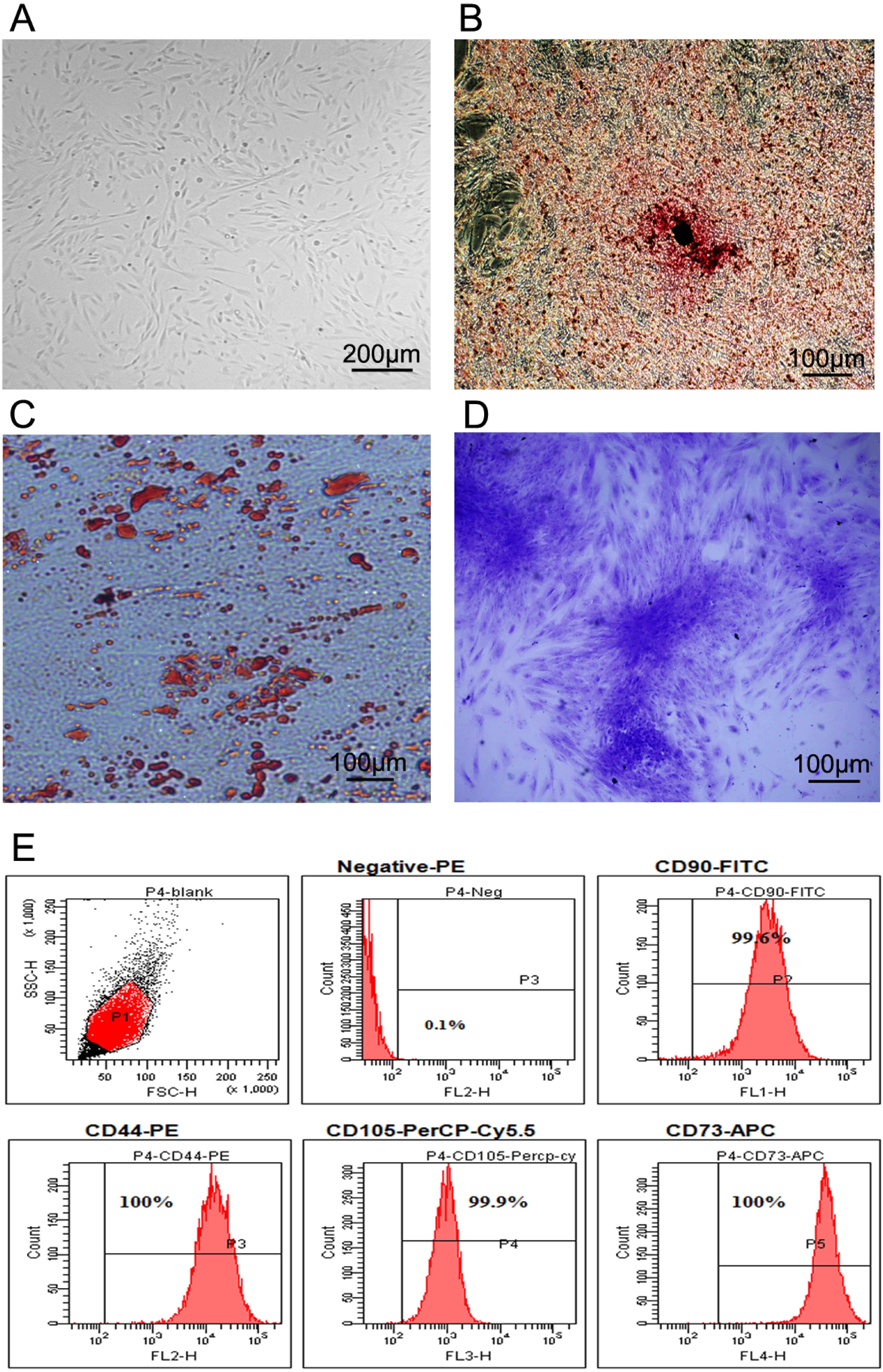

A
200μm
B
100μm
C
100μm
D
100μm
E
P4-blank
P1
SSC-H
FSC-H
(x 1,000)
Negative-PE
P4-Neg
P3
0.1%
Count
FL2-H
CD90-FITC
P4-CD90-FITC
99.6%
Count
FL1-H
CD44-PE
P4-CD44-PE
100%
P3
Count
FL2-H
CD105-PerCP-Cy5.5
P4-CD105-Percp-cy
99.9%
P4
Count
FL3-H
CD73-APC
P4-CD73-APC
100%
P5
Count
FL4-H

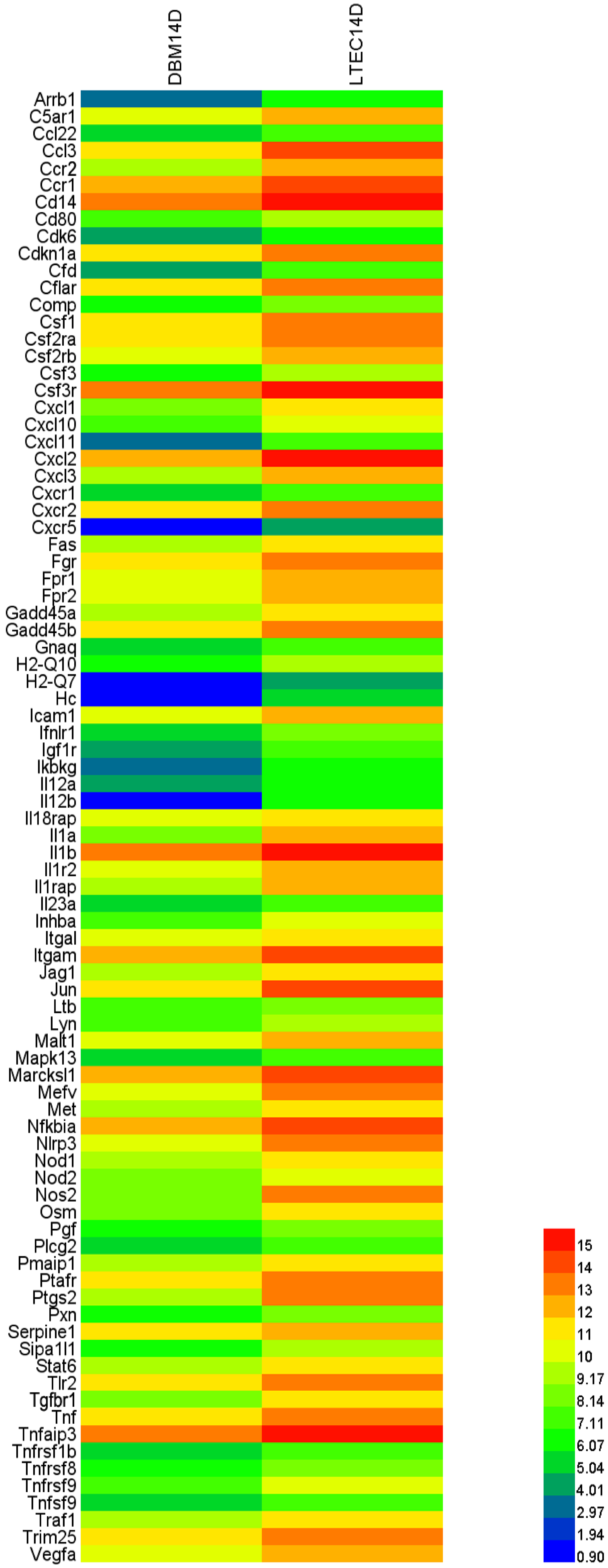

DBM14D
LTEC14D
Arrb1
C5ar1
Ccl22
Ccl3
Ccr2
Ccr1
Cd14
Cd80
Cdk6
Cdkn1a
Cfd
Cflar
Comp
Csf1
Csf2ra
Csf2rb
Csf3
Csf3r
Cxcl1
Cxcl10
Cxcl11
Cxcl2
Cxcl3
Cxcr1
Cxcr2
Cxcr5
Fas
Fgr
Fpr1
Fpr2
Gadd45a
Gadd45b
Gnaq
H2-Q10
H2-Q7
Hc
Icam1
Ifnlr1
Igf1r
Ikbkg
Il12a
Il12b
Il18rap
Il1a
Il1b
Il1r2
Il1rap
Il23a
Inhba
Itgal
Itgam
Jag1
Jun
Ltb
Lyn
Malt1
Mapk13
Marcksl1
Mefv
Met
Nfkbia
Nlrp3
Nod1
Nod2
Nos2
Osm
Pgf
Plcg2
Pmaip1
Ptafr
Ptgs2
Pxn
Serpine1
Sipa1l1
Stat6
Tlr2
Tgfbr1
Tnf
Tnfaip3
Tnfrsf1b
Tnfrsf8
Tnfrsf9
Tnfsf9
Traf1
Trim25
Vegfa
15
14
13
12
11
10
9.17
8.14
7.11
6.07
5.04
4.01
2.97
1.94
0.90

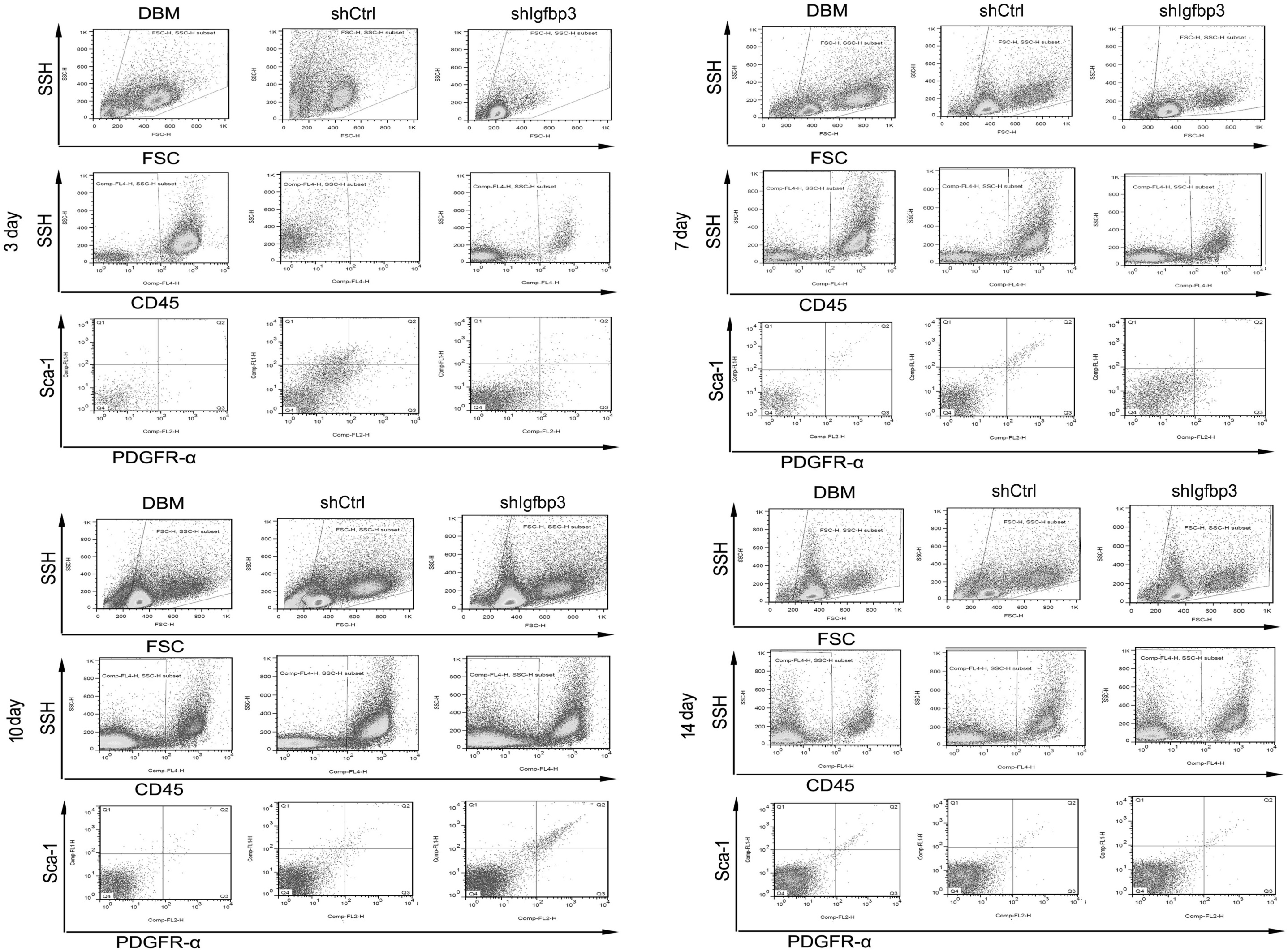

3 day
7 day
10 day
14 day
DBM
shCtrl
shIgfbp3
SSH
FSC
CD45
Sca-1
PDGFR-α
FSC-H, SSC-H subset
Comp-FL4-H, SSC-H subset
FSC-H
SSC-H
Comp-FL4-H
Comp-FL1-H
Comp-FL2-H
Q1
Q2
Q3
Q4

Supplemental Figure1 Identification of hUCMSCs. A: Representative phase images of adherent P4 MSCs. B: The presence of calcium mineralization (Alizarin Red). C: Lipid vacuoles formation (Oil red O). D: The presence of chondrogenesis (Alcian Blue). E: Phenotype of P4 MSCs (MSC positive cocktail: FITC CD90, PerCP-Cy™5.5 CD105 and APC CD73, and the negative MSC cocktail: PE CD45, PE CD34, PE CD11b, PE CD19 and PE HLA-DR)

Supplemental Figure 2 Heatmap constructed by promigratory 89 genes derived from the significant pathways.

Supplemental Figure 3: Endogenous MSC kinetics postimplantation. Representative Sca-1 versus PDGFR-α plots were gated on CD45- cells in DBM, *shCtrl* and *shIgfbp3* groups at 3, 7, 10 and 14 days postimplantation. Plots are representative of three independent experiments.
